# Star-Shaped and Linear POSS-Polylactide Hybrid Copolymers

**DOI:** 10.3390/ma8074400

**Published:** 2015-07-17

**Authors:** Krystyna Rozga-Wijas, Wlodzimierz A. Stanczyk, Jan Kurjata, Slawomir Kazmierski

**Affiliations:** Centre of Molecular and Macromolecular Studies, Polish Academy of Sciences, Sienkiewicza 112, 90-363 Lodz, Poland; E-Mails: was@cbmm.lodz.pl (W.A.S.); jkurjata@cbmm.lodz.pl (J.K.); kaslawek@cbmm.lodz.pl (S.K.)

**Keywords:** functionalized POSS, thiol-ene addition, star-shaped polymers, organo-inorganic hybrid polymers, polylactide

## Abstract

Novel octakis-2[(6-hydroxyhexyl)thio]ethyl-octasilsesquioxane (POSS-S-OH) as well as heptaisobutyl-2[(6-hydroxyhexyl)thio]ethyl-octasilsesquioxane (iBu-POSS-S-OH) were synthesized. POSS structures, bearing both types of groups *i.e.*, 2[(6-hydroxyhexyl)thio]ethyl and the vinyl ones, pendant from the octahedral cage are also described. The synthetic pathway involved thiol-ene click reaction of 6-mercapto-1-hexanol (MCH) to octavinyloctasilsesquioxane (POSS-Vi), and heptaisobutylvinyloctasilsesquioxane (iBu-POSS-Vi), in the presence of 2,2′-azobisisobutyronitrile. The functionalized silsesquioxane cages of regular octahedral structure were used further as initiators for ring opening polymerization of l,l-dilactide, catalyzed by tin (II) 2-ethylhexanoate. The polymerization afforded biodegradable hybrid star shape and linear systems with an octasilsesquioxane cage as a core, bearing polylactide arm(s).

## 1. Introduction

Polyhedral oligomeric silsesquioxanes (POSS) present a unique class of inorganic structures that can be exploited in generation of hybrid polymer systems of advantageous properties [1,2,3,4,5,6]. Among these, cube octameric silsesquioxanes (RSiO_1.5_)_8_ have been recently covalently bonded to peptide and glycoside moieties as well as such polymers as e.g., polylactide (PLA), poly(ethylene oxide) (PEO), glycomethacrylate or poly(caprolactone) (PCL) [2,7,8,9,10].

POSS-containing lactide polymers, including composites obtained by physical blending, have become a subject of increasing interest in recent years. It is expected, that linear and star-shaped POSS containing polymers can be used as valuable materials for biomedical applications, as both lactide blocks and POSS moieties are biodegradable [11,12,13,14,15,16].

Up to now a number of synthetic methods have been proposed, leading to various PLA-POSS hybrids. They include the use of mono-functionalized POSS (3-hydroxypropylheptaisobutyl and 2-hydroxyethylheptavinyl POSS) as initiators for lactide polymerization in the presence of stannous (II) octanoate, (Sn(Oct)_2_) as catalyst [17,18]. General methods of the functionalization of POSS molecules were reviewed by Kuo and Chang [19]. The metalla-silsesquioxane-hepta(isobutyl)-(isopropoxy-titanium)-silsesquioxane (Ti-POSS) was used as initiator of the ring-opening polymerization of lactide via a coordination-insertion mechanism [20]. Dubois *et al.* applied aminopropylheptaisobutyl POSS as an effective initiator in polymerization of lactide and formation of block copolymers with a polycaprolactone-*b*-polylactide chain [21]. Syntheses of POSS hybrid star systems, bearing eight chains of polylactide or polylactide-*b*-(poly(N-izopropylacrylamide) were initiated from POSS bearing octa(glicydyl ether) [22] and octa(3-hydroxypropyl) [12] moieties, respectively. This approach known as core-first method relies on initiation of polymerization by eight functional groups of POSS. The other method—Arm-first approach—Involves coupling of the pre-prepared linear polymers with the POSS core, affording homo- and hetero-arm star-shaped polymers [2,9]. In these cases octa(anhydride) [9] and octa(azide) [2], functionalized POSS were reacted, respectively, with modified polylactides bearing hydroxyl or alkyne moieties. 

Literature data, including our own results, point to hydroxyl and amino-functionalized POSS as the effective initiators of lactide and lactone polymerization [9,12,17,18,23,24]. Mono-functionalized hydroxyalkyl POSS molecules were typically made by a corner-capping reaction of incompletely condensed heptaisobutyl POSS trisilanols with e.g., hydroxyalkyltrichlorosilanes [18] or 2-(trichlorosilyl)ethyl acetate, followed by hydrolysis of the relevant ester and yielding mono-hydroxylalkyl derivatives of POSS [23]. POSS having other reactive functional groups e.g., Si–OR, Si–H and C=C can undergo suitable chemical transformations such as substitution and hydrosilylation, leading to a wide range of POSS functional systems [9,24,25].

Previously we proved that (3-mercaptopropyl)trimethoxysilane can react with vinyl groups at the silicon atoms of octavinyloctasilsesquixane to afford POSS having a variable number of alkoxysilyl functions [26]. This thiol-ene addition [27] serves as a convenient synthetic tool, particularly in the area of POSS materials [17,24,25,26,27,28,29,30,31]. This regioselective process, proceeds under mild conditions and tolerates many solvents, and functional groups. The method avoids metal catalysts, which could contaminate the addition product. The additional advantage of the thiol-ene addition is that it introduces thioether functions to the product, which may be further modified [32]. In most of the published work the thiol-ene click addition process involved reaction of thiols with octavinyl- or octavinyldimethylsilyloxy-POSS [33,34,35,36]. 

In this paper we present results of our studies on the synthesis of POSS-containing polymer hybrids, where the polymer chain was generated by ring-opening polymerization of l,l-dilactide monomer in the presence of POSS initiators, bearing one or eight 6-hydroxy-n-hexylthio-2ethyl groups. Such biodegradable POSS lactide systems can be extremely useful as nanocarriers in biomedical applications. Additionally, the use of multi-arm structures allows the conjugation of not only a therapeutic agent (drug) but also a targeting moiety (e.g., folic acid) and an imaging agent (e.g., fluorescein).

## 2. Results and Discussion

**Scheme 1:**
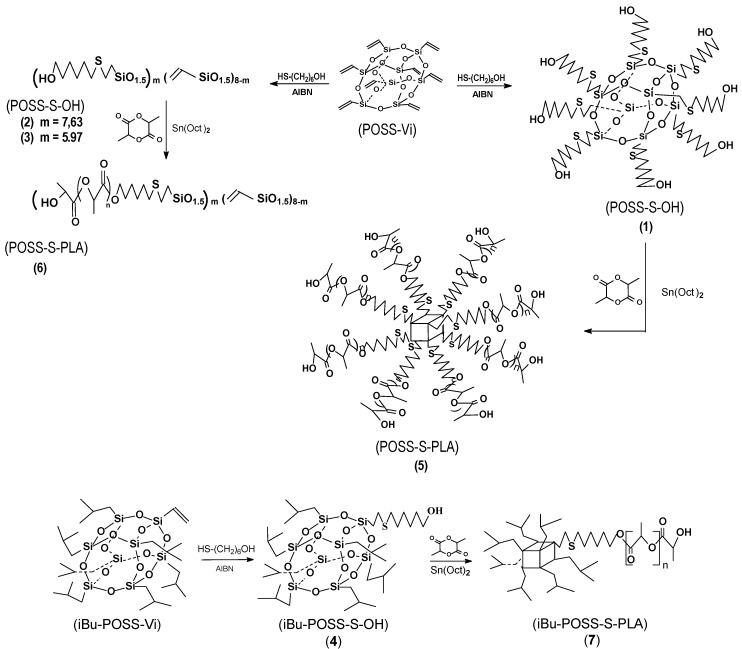
Routes to star-shaped and linear silsesquioxane-lactide hybrid systems.

Polymerization of l,l-dilactide was performed using octakis-2[(6-hydroxyhexyl)thio]ethyl-octasilsesquioxane (POSS-S-OH) or heptaisobutyl-2[(6-hydroxyhexyl)thio]ethyl-octasilsesquioxane (iBu-POSS-S-OH) in the presence of tin(II) 2-ethylhexanoate (Sn(Oct)_2_) catalyst. The initiating hydroxyl terminal groups were introduced by thiol-ene addition of 6-mercaptohexanol to octavinyloctasilsesquioxane (POSS-Vi) and vinylheptaisobutyloctasilsesquioxane (iBu-POSS-Vi) (Scheme 1). The 6-hydroxy-n-hexylthio-2-ethyl moiety was used as an initiating center in the synthesis of star-shaped and linear telechelic organo-inorganic materials. It was shown that the synthesis routes lead to a high yield of copolymers having a narrow molecular weight distribution.

### 2.1. Synthesis of Reactive Initiators—6-Hydroxy-n-Hexylthio-2-Ethyl Functionalized POSS.

We studied the thiol-ene addition of 6-mercapto-1-hexanol (MCH) to vinyl groups bound to silicon atoms of the silsesquioxane cage, initiated by 2,2′-azobisisobutyronitrile (AIBN). The general features of the mechanism of this free radical process were described earlier [26,36,37,38,39]. In all cases studied the addition occurs with high yield and high selectivity according to the anti-Markovnikov rule. The contribution from α addition is usually negligible (<3%) [39]. However, Li [40] claimed that no α-product was observed in UV initiated reactions of monovinyl substituted POSS with a series of thiols bearing hydroxyl, carboxyl and trialkoxysilane groups.

The addition reaction was carried out at 60 °C in toluene solution using 5% molar excess of MCH to ensure conversion of the vinyl groups. However, if vinylheptaisobutylocta-silsesquioxane (iBu-POSS-Vi) was used, the addition was less effective, with 92.54% conversion. The results are summarized in Table 1.

**Table 1:** Thiol-ene addition of 6-mercapto-1-hexanol to vinyl substituted cube-octameric silsesquioxanes.

Symbol of Silsesquioxane	Precursors	–CH=CH_2_/ HS(CH_2_)_6_OH [mol/mol]	Time of Reaction [min] ^(a)^	Conversion of Vinyl Groups ^(b)^ [mol%]	Sulfur Content [wt %] ^(c)^	Contents of R ^(d)^ [mmol/1 g]
1	POSS-Vi	1 : 1.05	240	99.45	15.06	4.70
2	POSS-Vi	1 : 1.05	150	95.37	14.79	4.62
3	POSS-Vi	1 : 0.53	420	74.60	13.01	4.06
4	iBu-POSS-Vi	1 : 1.05	240	92.54	3.24	1.01

(**a**) at 60 °C; (**b**) conversion of vinyl groups from ^1^H NMR, determined after purification of the product; (**c**) theoretical content of sulfur are 15.00% and 3.27% for POSS[(CH_2_)_2_S(CH_2_)_6_OH]_8_ and iBu_7_POSS[(CH_2_)_2_S(CH_2_)_6_OH] respectively; (**d**) contents of R = –(CH_2_)_2_S(CH_2_)_6_OH groups [mmol/g], based on microanalysis.

Partial addition of MCH leads to the POSS molecule bearing both functions— 6-hydroxy-*n*-hexylthio-2 ethyl and the vinyl ones. The average ratio of both functions in the POSS product can be easily controlled by the initial ratio of the substrates. The POSS adduct, having on average six 6-hydroxy-*n*-hexylthio-2-ethyl groups and two vinyl ones (3) was obtained with 68.21% yield, using a 1:2 initial molar ratio of MCH to vinyl groups in (POSS-Vi). The addition was carried out for a longer time (7 h) to achieve maximum conversion of MCH. It was confirmed by the absence of the thiol group resonance in ^1^H NMR at 1.29 ppm. It points to the possibility of a tailored synthesis of the polymer systems from an inorganic cube-octameric silsesquioxane core, having, on average, a desired number of functional groups capable to initiate polymerization.

#### 2.1.1. Spectroscopic Characterization of Functionalized POSS

**Figure 1:**
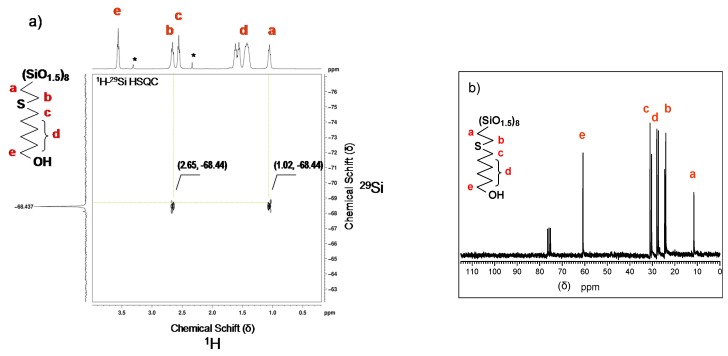
NMR spectra of octakis-2[(6-hydroxyhexyl)thio]ethyl-octasilsesquioxane, (POSS-S-OH)(1) (**a**) ^1^H–^29^Si HSQC in CD_3_OD, (*-solvents); (**b**) ^13^C NMR in CDCl_3_.

Progress of the thiol addition to the carbon-carbon unsaturated bond of octavinyloctasilsesquioxane was followed by ^1^H NMR. The disappearance of the resonance corresponding to the vinyl groups at 5.7–6.0 ppm in POSS is accompanied by creation of new resonances: at 1.02 ppm (t, SiCH_2_CH_2_) and two triplets centered at 2.55 and 2.65 ppm (CH_2_SCH_2_), as well as the triplet at 3.56 ppm (t, CH_2_OH). The relative intensity of these resonances equals 1, while the one corresponding to the “middle” (CH_2_) groups in the hexyl linker at 1.36–1.73 ppm, is four times more intense (Figure 1a and Figure S1 see supplementary materials). In the ^13^C NMR spectra a resonance corresponding to the formation of the Si–CH_2_ bond at 11.69 ppm is observed. There is also a signal at 60.81 ppm, which represents the resonance of the (CH_2_OH) carbon (Figure 1b).

After thiol-ene addition the POSS cage structure is preserved as can be seen from the relevant ^29^Si NMR spectrum of the addition product (Figure S2 see supplementary materials). There is only a single sharp resonance at –68.44 ppm, which represents eight silicon atoms bearing 2-[(6-hydroxyhexyl)thio]ethyl side groups (POSS-S-OH)(1), while the resonance for the POSS substrate at −80.2 ppm has disappeared. 

The structure of the product is further supported by ^1^H–^29^Si HSQC 2D NMR (Figure 1a). The two cross peaks appear in the 2D NMR spectrum of (POSS-S-OH)(1). One of them corresponds to the coupling between corner silicon atoms and the protons at α carbon (Si–C–H) at 1.02 ppm, while the other one relates to conjugation between silicon and protons at β carbon atom (Si–C–C–H) at 2.65 ppm. The values of coupling constants ^2^J(Si–H) = 11 Hz and ^3^J(Si–H) = 9 Hz were calculated from the ^1^H–^29^Si HMBC 2D NMR spectrum.

**Figure 2:**
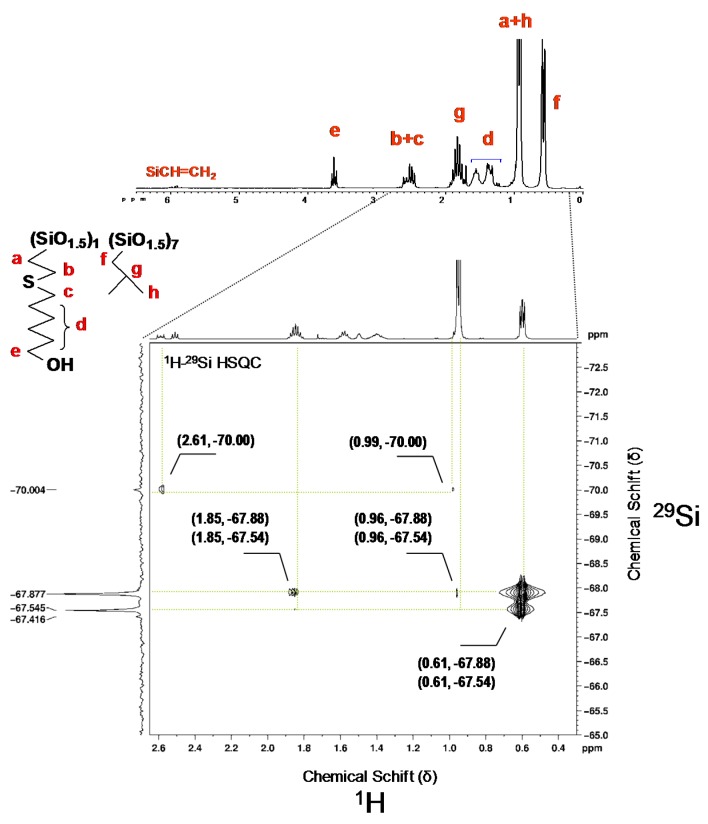
^1^H–^29^Si HSQC(CDCl_3_) spectrum of (iBu-POSS-S-OH)(4) obtained by thiol-ene addition of 6-mercaptohexanol-1 to heptaisobutylvinyloctasilsesquioxane (conversion of vinyl groups 92.54%).

In the case of 2[(6-hydroxyhexyl)thio]ethyl-heptaisobuthyloctasilsesquioxanes (iBu-POSS-S-OH)(4), a careful ^1^H NMR analysis has shown a characteristic doublet at 0.96 ppm of methyl protons in isobutyl groups, a doublet of methylene protons at 0.61 ppm, and a multiplet of methine proton at 1.74–1.92 ppm. The resonance for newly created Si–CH_2_ bond can be identified at 0.99 ppm. The most diagnostic resonances appear as a triplet at 3.65 ppm (CH_2_OH) and as two equal multiplets at 2.52 and 2.61 ppm (CH_2_SCH_2_). A small resonance, originating most probably from traces of vinyl groups of the POSS substrate is also visible, at 5.85–6.07 ppm (Figure 2), however the resonances corresponding to vinyl groups are missing from the relevant ^13^C and ^29^Si spectra (Figures S3 and S4 see supplementary materials).

An additional confirmation of the (iBu-POSS-S-OH)(4) structure is provided by the ^29^Si NMR spectrum, which shows new resonance for silicon bearing 2[(6-hydroxyhexyl)thio]ethyl group at −70.00 ppm (Figure 2 and Figure S5).

In the ^1^H–^29^Si NMR spectrum the signals originating from silicon atoms, in various surrounding in the octahedral cage, which are bound to isobutyl substituents are clearly visible at −67.87 and −67,54 ppm. The almost complete disappearance of the resonance at −80.20 ppm (Si–CH=CH_2_) was accompanied by generation of the new one at −70.00 ppm, corresponding to mercaptohexanol addition to the vinyl group (Figure 2). The recrystalized (iBu-POSS-S-OH)(4) contained about 7.5% of unreacted heptaisobutylvinyl POSS.

The silicon atom bearing 6-hydroxy-n-hexylthio-2-ethyl moiety couples with four hydrogen atoms. At 2.61 ppm there is a strong coupling across three bonds as well as another one at 0.99 ppm across two bonds. The spectrum allows also for identification of weak Si-H coupling across four bonds of the silicon atoms bearing isobutyl substituents (six CH_3_ protons) at 0.96 ppm. In the standard ^1^H NMR spectrum the resonances for the CH_3_ group (in Si–iBu) and the bridging CH_2_ in (SiCH_2_CH_2_S) overlap and do not allow for a quantitative evaluation of the conversion of (SiCH=CH_2_) moieties in the substrate. The two Si resonances, correspond to silicon atoms in different surroundings bound to iBu substituents. Silicon atoms couple with (SiCH_2_) protons across two bonds at 0.61 ppm and (SiCH_2_CH) proton at 1.8 ppm across three bonds.

#### 2.1.2. MALDI-TOF Mass Spectra of 6-Hydroxy-*n*-Hexylthio-2-Ethyl Functionalized POSS (Macroinitiators for Polymerization of LA)

**Figure 3:**
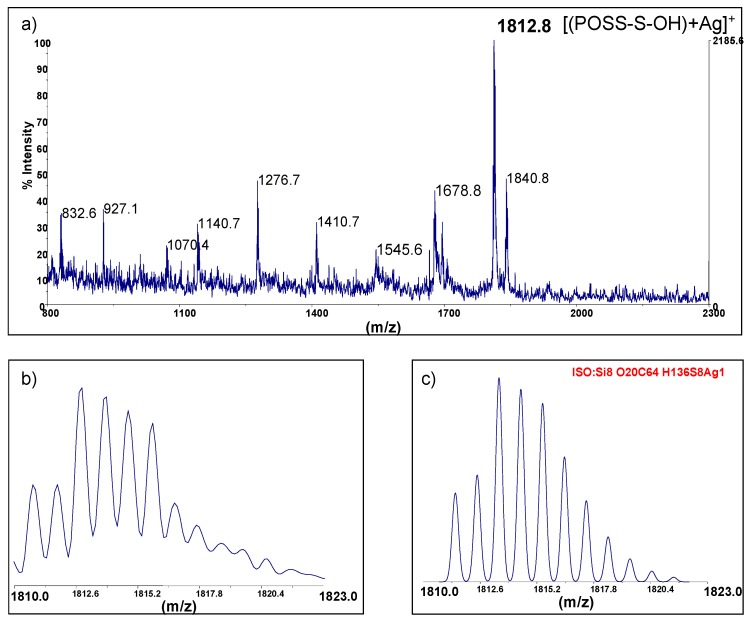
MALDI-TOF spectra (linear mode, AgTFA added) of the (POSS-S-OH)(1), C_64_H_136_O_20_S_8_Si_8_, 1706.44. (**a**) containing 99.45% of reactive –(CH_2_)_2_S(CH_2_)_6_OH groups; (**b**) expansion of the 1810–1823 *m*/*z* range; (**c**) simulated spectrum.

An exemplary MALDI TOF spectrum of (POSS-S-OH)(1) is shown in Figure 3. The main peak (*m*/*z* = 1812.8) corresponds to the [M + Ag]^+^ of totally substituted POSS. Isotopic pattern of this signal consists of ten lines in a range of *m*/*z* = 1810–1823 and their shape and mutual relation is identical to the result of computer simulation for C_64_H_136_O_20_S_8_Si_8_, based on isotope composition. The two other signals point to the presence of partly reacted silsesquioxanes having respectively one and two vinyl substituents at *m*/*z* = 1678.8–[POSS-{CH_2_CH_2_S(CH_2_)_6_OH}_7_(CH=CH_2_) + Ag]^+^ and *m*/*z* = 1545.6–[POSS-{CH_2_CH_2_S(CH_2_)_6_OH}_6_(CH=CH_2_)_2_ + Ag]^+^ .

**Figure 4:**
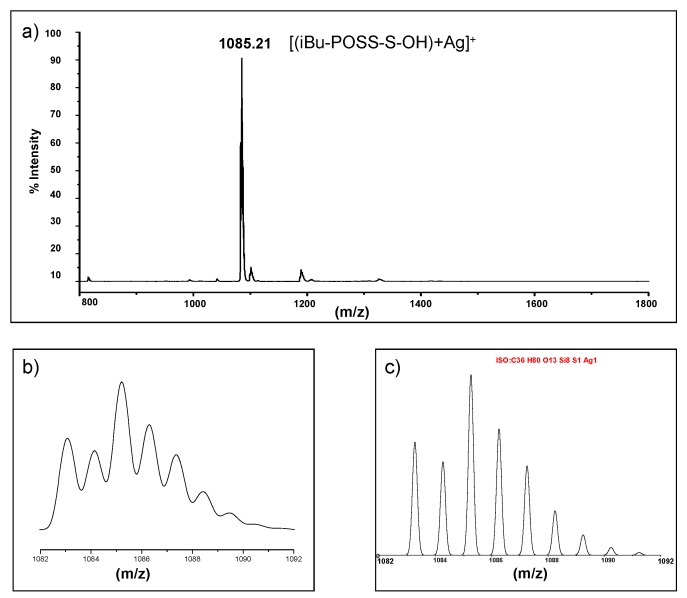
MALDI-TOF spectra (linear mode, AgTFA added) of the (iBu-POSS-S-OH)(4), C_36_H_80_O_13_S_1_Si_8_, *M* = 977.70. (**a**) containing 92.54% of reactive –(CH_2_)_2_S(CH_2_)_6_OH groups; (**b**) expansion of 1082–1092 *m*/*z* range; (**c**) simulated spectrum.

MALDI-TOF mass analysis performed for POSS derivative with one 2-[(6-hydroxyhexyl) thio]ethyl group and seven isobutyl groups (iBu-POSS-S-OH)(4) exhibited only one main signal for [(iBu-POSS-S-OH) + Ag]^+^ (Figure 4). Isotopic pattern of this signal corresponds to the simulated spectrum in a range of *m*/*z* = 1082–1092 for C_36_H_80_O_13_S_1_Si_8_, basing on isotope composition.

### 2.2. Synthesis of Star-Shaped and Linear POSS–Lactide Hybrids

Octakis-2[(6-hydroxyhexyl)thio]ethyl-octasilsesquioxane (POSS-S-OH) and heptaisobutyl-2[(6-hydroxyhexyl)thio]ethyl-octasilsesquioxane (iBu-POSS-S-OH) were used as initiators in the Ring Opening Polymerization (ROP) of l,l-dilactide.

The mechanism of the ROP of cyclic esters initiated by tin (II) 2-ethylhexanoate and primary alcohols has been studied and reported in the literature [41,42,43]. The polymerization proceeds through a coordination-insertion mechanism involving the O-acyl cleavage of the cyclic l,l-dilactide [44]. In the presence of the hydroxyl group, linear polymers terminated with a secondary hydroxyl group are formed. The molecular weight can be controlled by adjusting the ratio of monomer to initiator.

Polymerization was carried out in dry, deoxygenated toluene, using tin (II) 2-ethylhexanoate catalyst at 110 °C, at the monomer/catalyst molar ratio of 300:1. Copolymers were characterized by FT-IR (Figure S6), ^1^H, ^13^C, ^29^Si NMR, SEC and MALDI TOF mass spectrometry. The experimental conditions and results are presented in Table 2. The use of POSS reagent containing eight (6-hydroxyhexyl)thio]ethyl groups led to the formation of a star-shaped silsesquioxane-polylactide hybrid (POSS-S-PLA), while the mono functional silsesquioxane initiator generated a linear system(iBu-POSS-S-PLA).

**Figure 5:**
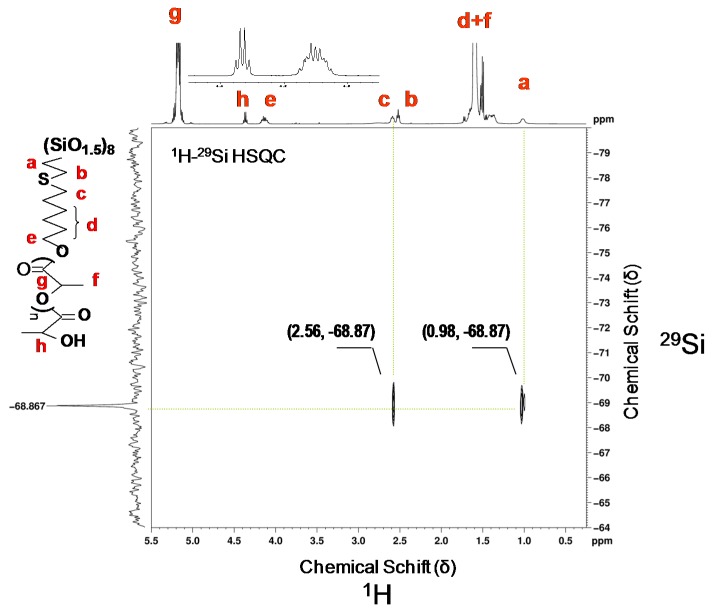
The ^1^H–^29^Si HSQC NMR spectrum of star-shaped (POSS-S-PLA)(5) obtained by polymerization of L,L-dilactide from octakis-2[(6-(hydroxyhexyl)thio]ethyl-octasilsesquioxane (1) catalyzed by tin (II) 2-ethylhexanoate. Molecular weight of the hybrid *M*_n_ = 28,500, PDI = 1.15 and *M*_n_ of polylactide chain *M*_n_ = 3300.

Conversion of l,l-dilactide was determined by running ^1^H NMR spectra of the polymerization mixture. After 24 h at 110 °C the methine proton (CH) resonance of l,l-dilactide monomer at –5.01 ppm became negligible compared with the equivalent methine ^1^H NMR resonance at 5.19 ppm of lactide units in the polymers. The relevant NMR spectra are shown in Figure 5 and Figure S7.

**Table 2:** Synthesis of star-shaped (POSS-S-PLA) and linear (iBu-POSS-S-PLA) hybrids.

Hybrid Polymers	Initiator Used	Content of R ^(a)^ in POSS Molecule		[LA]/[OH] [mol/mol]	Polymerization	
		[mol] % ^(b)^	[mmol/g] ^(c)^		Conversion of LA% ^(b)^	Yield%
5	POSS-S-OH (1)	99.45	4.66	22.78	94.05	91.70
6	POSS-S-OH (2)	95.37	4.47	9.91	84.47	82.40
7	iBu-POSS-S-OH (4)	92.54	0.95	34.74	78.9	73.51

(**a**) R = –(CH)_2_S(CH_2_)_6_OH; (**b**) from ^1^H NMR; (**c**) from microanalysis.

#### 2.2.1. SEC Analysis

**Figure 6:**
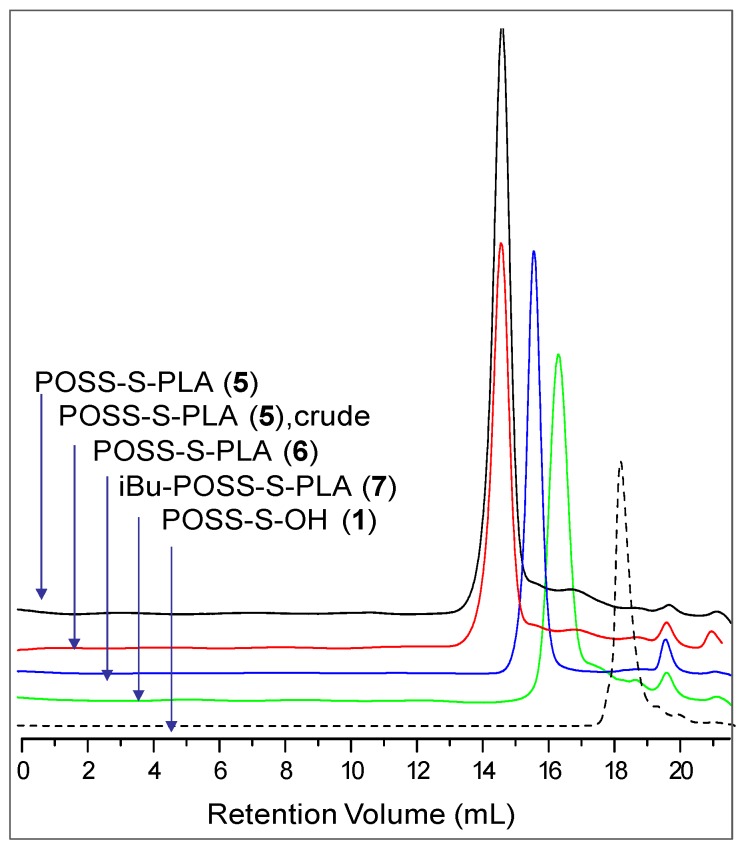
SEC traces of star-shaped (POSS-S-PLA)(5), *M*_n_(MALS) = 28,500, PDI = 1.15 before precipitation—red line, after precipitation in methanol—black line, (6) *M*_n_(MALS) = 9050, PDI = 1.07—blue line and (iBu-POSS-S-PLA)(7) *M*_n_(MALS) = 3600, PDI = 1.04—green line. Initiator (POSS-S-OH)(1)—black dashed line.

Molecular weights determined from SEC, using a refractive index (RI) detector, based on polystyrene standards, were much higher than expected from the monomer/initiator ratio (Table 3, Column 2) as a result of a different hydrodynamic behavior of the studied star-shaped macromolecule, compared to the standard. A dual detector combining tandem multiangle light-scattering MALS and RI detectors coupled with a size exclusion chromatograph, was used to evaluate the absolute values of the average molecular weight (Table 3, Column 6) [45]. The ROP polymerizations of l,l-dilactide initiated by eight hydroxyalkyl-functionalized silsesquioxane allowed the achievement of a conversion of l,l-dilactide in the range of 80%–95%. Size-exclusion chromatograms revealed monomodal peaks of the star-shaped POSS containing polymers with exceptionally narrow molecular weight distribution of 1.15 and 1.06, respectively for copolymers (5) and (6). The *M*_n_ of 28,500 for hybrid (POSS-S-PLA)(5) from MALS means that each Si atom in the octahedral cage is grafted with a linear PLA polymer chain having on average 23 monomeric units. The molecular weight of one statistical polylactide chain equals 3340. Results of the NMR and SEC analyses are summarized in Table 3 and the relevant size exclusion chromatograms of the POSS-lactide hybrids are presented in Figure 6. The narrow molecular weight distribution obtained in these experiments is not a result of the final purification process as the same PDI of 1.15 is observed before and after the precipitation in methanol (Figure 6).

The star-shaped polymer with shorter chains (6) showed an even lower PDI (1.07), (Figure 6). The average number of arms grown from the POSS cage (6), was calculated as 7.63 per cube (*M*_n_ = 9050). Each of the arms consists on average of 7–8 monomeric units. The molecular weight of the lactide chain grafted from the single site of octakis-2[(6-hydroxyhexyl)thio]ethyl-octasilsesquioxane (POSS-S-OH)(2) was equal to 960 (SEC) and 1060 (^1^H NMR) compared to the theoretical value of 1210. 

**Table 3:** Polymers of l,l-dilactide initiated from POSS structures.

Hybrid Polymers	Mn of Hybrids					PDI		Number of Chains per POSS	Mn of Chain/ DP		
	Theor.	Micr. Anal.	NMR	RI ^(a)^	MALS	RI	MALS		Theor. ^(b)^	NMR ^(c)^	MALS
5	26,350	28,430	34,000	32,000	28,500	1.15	1.15	8	3,080/21.4	3,170/22	3,340/23
6	11,400	–	9,800	13,400	9,050	1.06	1.07	7.63	1,210/8.4	1,060/7.4	960/6.7
7	4,960	4,600	4,550	4,950	3,600	1.28	1.04	1	3,980/27.6	3,570/25	2,600/18.2
POSS–S–OH (1)	1,706.4	–	–	–	1,820	–	1.007	–	–	–	–
iBu–POSS–S–OH (4)	977.7	–	–	–	1,085	–	1.14	–	–	–	–

(**a**) From SEC (relative to polystyrene standards); (**b**) *M*_nTheor_ = ([m_LA_]_o_/[(CH)_2_S(CH_2_)_6_OH]) × conv.; (**c**) from ^1^H NMR in CDCl_3_.

#### 2.2.2. NMR Analysis of the Hybrid Polymers 

The ^1^H NMR spectra confirm the covalent grafting of the polylactide chains, initiated from functionalized silsesquioxanes. The triplet resonance characteristic of (CH_2_OH) protons at 3.56 ppm in (1) shifts to 4.11 ppm due to the formation of a new ester bond (CH_2_OC(O)) in the copolymer, while the small quartet resonance at 4.33 ppm originates from methine protons (CH) at the end of the polylactide chains. The structure of the polymer hybrid is further proven by resonances characteristic of the [–CH_2_CH_2_S(CH_2_)_6_O-] spacer at 0.98 ppm (SiCH_2_), 1.25–1.74 ppm (SCH_2_(CH_2_)_4_), and multiplets at 2.48 (SCH_2_(CH_2_)_4_), 2.56 (SiCH_2_CH_2_S) ppm, and 4.11 ppm ((CH_2_)_4_CH_2_O) (Figure 5).

The number of lactide monomeric units in the star hybrid polymer was calculated from the ratio of the integrals of the (CH_2_OC(O)) methylene protons which appear at 4.11 ppm to the (SiCH_2_) ones (0.98 ppm) at the silsesquioxane core. Similarly, the chain length of the polylactide arms was calculated from the integration ratio of CH resonance in the lactide chain at 5.16 ppm to the terminal CH resonance at 4.33 ppm and to the (CH_2_OC(O)) protons at 4.11 ppm. The absence of resonances for the (CH_2_OH) functional group of silsesquioxane at 3.56 ppm indicated that all them initiated polymerization (Figure 5 and Figure S8 see supplementary materials).

In the ^1^H–^29^Si NMR spectrum of the (POSS-S-PLA) star-shaped system (5) (Figure 5) there is only one strong resonance at –68.87 ppm, representing eight silicon atoms of the POSS cage. It exhibits the same shift as the silicon atoms in the (POSS-S-OH) initiator, pointing out that the polymerization does not affect the chemical shift of the cage silicon atoms. The spectrum reveals weak coupling across two and three bonds between silicon and (Si–C–H) and (Si–C–C–H) protons ^2^J(Si–H) = 15 Hz and ^3^J(Si–H) = 7 Hz, respectively.

**Figure 7:**
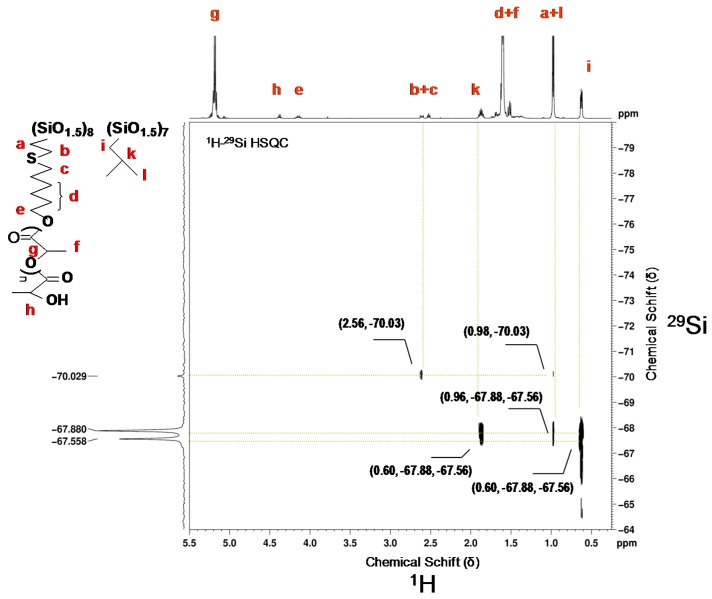
The ^1^H–^29^Si HSQC NMR spectrum of star-shaped (iBu-POSS-S-PLA)(7) obtained by polymerization of l,l-dilactide from (iBu-POSS-S-OH)(4) catalyzed by tin (II) 2-ethylhexanoate. Molecular weight of hybrid *M*_n_ = 3600, PDI = 1.04.

The ^1^H NMR spectrum of the linear hybrid (iBu-POSS-S-PLA)(7) (Figure 7) shows a new multiplet at 4.11 ppm corresponding to formation of the ester moiety (CH_2_OC(O)). The molecular weight (3570) of the single site polylactide chain in the POSS based polymer hybrid (linear system), calculated from the ratio of methine protons at 5.14 ppm in the polymer chain to the one at the end of the lactide chain at 4.33 ppm and to (CH_2_S) protons at 2.56 ppm was close to the theoretical value of 3980.

#### 2.2.3. MALDI TOF of Polymer Hybrids

**Figure 8:**
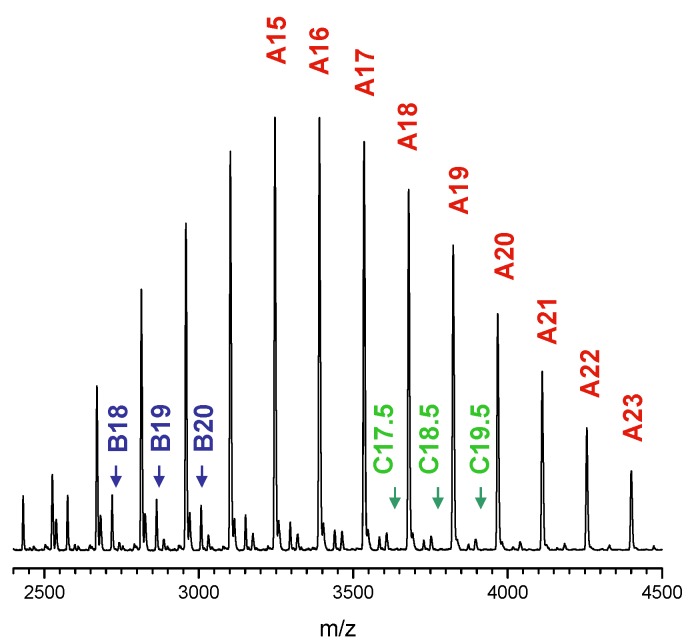
MALDI TOF spectrum of (iBu-POSS-S-PLLA)(7) hybrid obtained by polymerization of l,l-dilactide from (iBu-POSS-S-OH) catalyzed by tin (II) 2-ethylhexanoate, *M*_n_ = 3600, PDI = 1.04.

Maldi TOF spectra confirm additional proof for the structure of the synthesized hybrid systems (both star-shaped and linear). For example the spectrum of (iBu-S-POSS)(7) initiated hybrid corresponds well to the linear polylactide structure grown from functionalized POSS cage (Figure 8). Three series of molecular ions are observed. Series A shows molecular ions [iBu-POSS-S-PLA + Ag]^+^ that differ by the mass of lactide monomeric units grafted from the (iBu-POSS-S-OH) cage. Series B, of a very low intensity, represents pure lactide macromolecules initiated by traces of water in the reaction system, that typically in this polymerization is catalyzed by tin (II) 2-ethylhexanoate [46]. The last one, series C, again of a very low intensity, corresponds to the POSS-lactide hybrids containing lactic acid [OCH(CH_3_)C(O)] moieties that were formed via trans-esterification and show an odd number of acid moieties.

**Figure 9:**
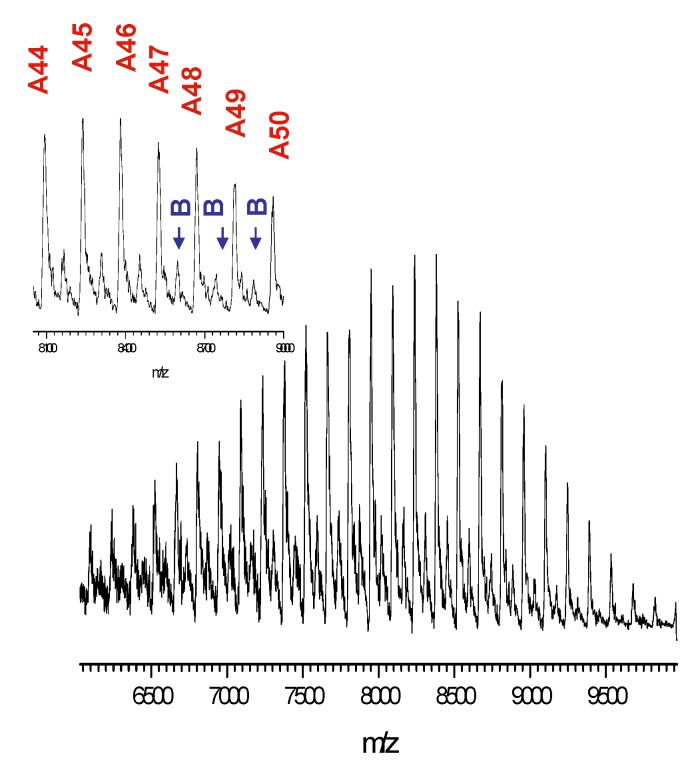
MALDI TOF spectrum of (POSS-S-PLLA)(6) hybrid obtained by the polymerization of l,l-dilactide from (POSS-S-OH) catalyzed by tin (II) 2-ethylhexanoate(2), *M*_n_ = 9050, PDI = 1.07.

The MALDI TOF spectrum of (POSS-S-PLLA)(6), *M*_n_ = 9 050 (Figure 9) shows two series of polymer hybrids. Series A corresponds to the star shape system initiated by functionalized POSS, while the low intensity (B) one is a result of the presence of macromolecules initiated by water. The extent of transesterification process is limited as the PDI is close to 1 [1.06 (IR) and 1.07(MALS)] (Table 3). MALDI TOF spectrum of (POSS-S-PLLA) No (5) could not be run as the Mn of 28500 exceeds the operating range of the spectrometer.

#### 2.2.4. Thermogravimetric Analysis

Thermogravimetric analysis (TGA) of 6-hydroxy-n-hexylthio-2-ethyl functionalized POSS and polylactide-POSS hybrids were performed under nitrogen in order to minimize reaction with air and moisture. Compounds (macroinitioators) (1) and (4) were found to undergo organic mass loss yielding permanent residues of 31% and 0.2% respectively. The onset temperatures for octakis-2[(6-hydroxyhexyl)thio]ethyl-octasilsesquioxane (1) (350 °C), is higher than that for heptaisobutyl-2[(6-hydroxyhexyl)thio]ethyl-octasilsesquioxane (4) (260 °C), as listed in Table 4.

**Table 4:** Thermal properties of organic-inorganic hybrid and hybrid polymers with POSS core.

Sample	*T*_onset_^(a)^ [°C]	*T*_max1_^(b)^ [°C]	*T*_max2_^(b)^ [°C]	Residue wt %
(POSS-S-OH)(1)	350	374	460	31.31
(iBu-POSS-S-OH)(4)	250	316	348	0.18
(POSS-S-PLA)(5)	192	232	389	3.19
(POSS-S-PLA)(6)	192	225	395	8.16
(iBu-POSS-S-PLA)(7)	210	231	317	3.51
Bu-PLA ^(**c**)^(8)	237	277	–	0.02

(**a**) *T*_onset_ is the onset decomposition temperature; (**b**) *T*_max1_ is the temperature corresponding to the maximum rate of the weight loss; (**c**) Bu-PLA (8), *M*_n_(RI) = 17800, PDI = 1.4.

**Figure 10:**
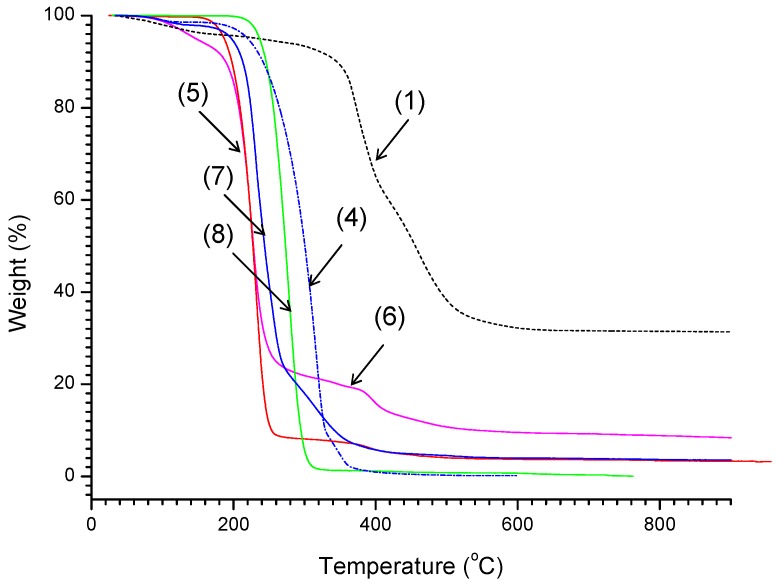
TGA traces of (POSS-S-OH)(1), (iBu-POSS-S-OH)(4) silsesquioxane and (POSS-S-PLA)(5) polylactides star polymer hybrid with an 8 arm length of 23 repeat units (*M*_n_ = 28,500), (POSS-S-PLA)(6) polylactides star polymer hybrid with an 7.63 arm length of 7 repeat units (*M*_n_ = 9050), (iBu-POSS-S-PLA)(7) linear hybrid (*M*_n_ = 3600) and Bu-polylactide polymer (*M*_n_ = 17,800, PDI = 1.4) (8).

The polylactide-POSS star-shaped hybrids (5) and (6) exhibit similar degradation profiles with the onset temperatures of 192 °C, indicating that the presence of POSS does not significantly alter the degradation when compared to model Bu-PLA polymer, initiated from n-butanol (onset temperature at 237 °C) Figure 10. In the case of the linear polylactide-POSS hybrid (7) the onset temperature of 210 °C is lower than the one obtained for Bu-PLA polymer, due to a shorter polymer chain. As far as residues are regarded, they appear to be related to the structure of the relevant polymers and oligomers [47]. While the structure is purely organic (8), the residue is the lowest. It increases for POSS containing copolymers with decreasing contents of organic component (PLA) and the decrease of its molecular weight. Linear copolymers give lower residue when compared to star-shaped systems.

## 3. Experimental Section 

### 3.1. Materials

All reactions were carried out under argon atmosphere. Octavinyloctasilsesquioxane (POSS-Vi, 97%) and vinylheptaisobutyloctasilsesquioxane (iBu-POSS-Vi, 97%) (Hybrid Plastics) were used without further purification. Toluene and dioxane (POCh) (analytical grade), were purified according to the standard methods [48] and kept under nitrogen. l,l-dilactide (LA) (Purac Biochem Company) was purified by recrystallization from toluene and dried at 60 °C under high vacuum for 12 h. The 6-mercapto-1-hexanol (MCH) (99%) and 2,2′-azobisisobutyronitrile (AIBN) (Aldrich) were used as supplied. Tin (II) 2-ethylhexanoate (Sn(Oct)_2_), 95%, was fractionally distilled before use. Silica gel (Merck grade 7734, pore size 60 Å, 70–230 mesh), methanol, tetrahydrofuran, and dichloromethane (POCh), analytical grade, were used as received.

### 3.2. Methods

Microanalysis was performed using an Euro EA elemental analyzer (Euro Vector Instruments & Software). Thermogravimetric analysis (TGA) was carried out with a Hi-Res TGA 2950 thermal analyzer. The measurements were carried out under nitrogen at the heating rate of 10 °/min from 30 °C to 960 °C. 

All the NMR spectra were run on a Bruker a AVANCE III DRX-500 MHz spectrometer, operating at 500.13, 125.77, and 99.36 MHz for ^1^H, ^13^C and ^29^Si, respectively. The spectrometer was equipped with 5 mm inverse broadband dual channel probe head with Z-gradients. All measurements were carried out at 295 K and the temperature was stabilized with a Bruker BCU 05 cooling system controlled by VTU 3200 unit. Spectra were acquired and processed with TopSpin 3.1 Bruker software running under Windows 7 platform. For all spectra, the chemical shift values were referenced externally to TMS (δ = 0.00 ppm for ^1^H, ^13^C and ^29^Si).

The quantitative 1D ^29^Si NMR spectra were obtained using the inverse-gated decoupling sequence. A 30° observe pulse was applied (90° pulse length was 15-μs) and the relaxation delay was set to 20 s.

The 2D ^1^H–^29^Si–HSQC spectra were acquired with 4096 points in F2 frequency domain and 256 increments. The delays for double inept transfer were optimized for J(Si–H) = 15 Hz. Acquired spectra were processed to 2K × 1K data points matrix and sine-bell apodisation phase-shifted 90° was applied prior to Fourier transformation.

Matrix-assisted laser desorption/ionization time-of-flight (MALDI-TOF) mass analysis was performed with a Voyager-Elite (PerSeptive Biosystems, Framingham, MA, USA) instrument equipped with N_2_ laser operating at 337 (pulse rate of 10 Hz) with positive polarity in the linear mode. The accelerating voltage was of 20 kV. The irradiation targets were prepared from THF solutions with DT, 1,8-dihydroxy-9(10H)-anthracenone, (dithranol) as matrix and silver trifluoroacetate (AgTFA) as a cationating agent (weight ratio of polymer:matrix:salt was equal to 1:1:0.1).

Size exclusion chromatographic analyses (SEC) for the synthesized polymers were run on a Wyatt Technology Corp. instrument equipped with Agilent Technologies pumps and Wyatt Optilab T-rex RI and MALS DAWN Heleos II laser photometer detectors. The laser operated at a wavelength of 632.8 nm. Samples were injected onto the battery of two PLgel Mixed-C columns: with 5 µm particle size. The eluent was dichloromethane at a flow rate of 0.8 mL/min. Dual detector MALS/RI SEC analysis including dn/dc determination was performed using ASTRA v. 6.1 software (Wyatt), assuming 100% mass recovery. The calibration constant value for the RI detector was determined separately, according to the procedure recommended by the producer.

### 3.3. Synthesis of Functionalized POSS

#### 3.3.1. Octakis-2[(6-Hydroxyhexyl)thio]ethyl-Octasilsesquioxane (POSS-S-OH)(1) 

Octakis-2[(6-hydroxyhexyl)thio]ethyl-octasilsesquioxane (POSS-S-OH)(1) was synthesized by modification of the general method described earlier in [26]. In a flask fitted with nitrogen inlet octavinyloctasilsesquioxane (POSS-Vi) (Figure S9) (1.6 g, 2.53 × 10^−3^ mol) and AIBN (0.017 g, 1 × 10^−4^ mol) were placed and 4 mL of toluene was introduced. The mixture was heated gently to 40 °C, while 6-mercapto-1-hexanol (MCH) (2.85 g, 21.25 × 10^−3^ mol) was added drop-wise. The reaction mixture was stirred at 60 °C for 4 h, to reach complete conversion of vinyl groups controlled by ^1^H NMR. The reaction mixture was cooled down to room temperature and evaporated and dried under vacuum for 14 h. The product was isolated after extraction of the excess of MCH and catalyst with toluene, evaporation of the solvent and dried under high vacuum (yield 4.32 g, 98.5%) of octakis-2[(6-hydroxyhexyl)thio]-ethyl-octasilsesquioxane (1). ^29^Si NMR (CD_3_OD) δ(ppm): –68.44 [s, (–O)_3_Si–CH_2_]. ^1^H NMR (CD_3_OD) δ(ppm): 1.02 (t, 16H, CH_2_–Si), 1.36–1.73 (m, 64H, –CH_2_–alkyl), 2.55 (t, 16H, CH_2_–S), 2.65 (t, 16H, S–CH_2_CH_2_–Si), 3.56 (m, 16H, CH_2_–OH). ^13^C NMR (CDCl_3_) δ(ppm): 11.58 (CH_2_–Si), 27.36 (S–CH_2_), 30.37 (CH_2_–S), 24.04, 24.50, 28.03, 31.02 (4 × –CH_2_), 60.81 (CH_2_OH). Anal. Found: C, 44.52; H, 7.91; S, 15.06. C_64_H_136_O_20_S_8_Si_8_ Calcd.: C, 45.05; H, 8.03; S, 15.00.

#### 3.3.2. Octakis-2[(6-Hydroxyhexyl)thio]ethyl-Octasilsesquioxane (POSS-S-OH)(3) 

Octakis-2[(6-hydroxyhexyl)thio]ethyl-octasilsesquioxane (POSS-S-OH)(3) bearing both (6-hydroxyhexyl)thioethyl and vinyl functions was obtained by reaction of (POSS-Vi) (2.0 g, 3.16 × 10^−3^ mol) and MCH (1.7 g, 12.64 × 10^−3^ mola) in 5 mL of toluene and AIBN (0.017 g, 1 × 10^−4^ mol), using an initial MCH to (POSS-Vi) molar ratio of 4:1. The addition was carried out for 7 h to achieve full conversion of MCH. The reaction mixture was concentrated and the solid product was washed with 80 mL of toluene separated and dried under vacuum. The crude sample was further purified by flash column chromatography (silica gel, with THF as the eluent) to afford the final product (1.84 g, 68.21%), conversion of vinyl group 74.60%. ^29^Si NMR (CD_3_OD) δ(ppm): –68.44 [s, (–O)_3_Si–CH_2_], –80.2 [s, (–O)_3_Si–CH=CH_2_]; ^1^H NMR (CD_3_OD) δ(ppm): 1.02 (t, CH_2_–Si), 1.30–1.75 (m, (–CH_2_–)_4_), 2.49–2.80 (m, CH_2_–S ), 3.61 (t, CH_2_–OH), 5.85–6.07 (m, CH=CH_2_); ^13^C NMR (CDCl_3_) δ(ppm): 12.08 (CH_2_–Si), 27.36 (S–CH_2_), 30.37 (CH_2_–S), 24.11, 24.51, 28.03, 31.08 (4 × –CH_2_), 60.85 (CH_2_OH), 135.1 and 136.3 .((-O)_3_Si–CH=CH_2_). Anal. Found: C, 42.63; H, 7.24; S, 13.01. 

#### 3.3.3. Preparation of 2[(6-Hydroxyhexyl)thio]ethyl-Heptaisobutyl-Octasilsesquioxane (iBu-POSS-S-OH)(4)

Preparation of 2[(6-hydroxyhexyl)thio]ethyl-heptaisobutyl-octasilsesquioxane (iBu-POSS-S-OH)(4) Vinylheptaisobutyl-POSS, (iBu-POSS-Vi), (1.4 g, 1.66 × 10^−3^ mol) and AIBN (0.017 g, 1 × 10^−4^ mol), MCH (0.24 g, 1.79 × 10^−3^ mol) and toluene (4 mL) were placed in a reaction flask, and kept at 60 °C for 4 h. After cooling down to room temperature, the solvent was removed in vacuum and the product was dissolved in CH_2_Cl_2_ and then precipitated in methanol. This purification procedure was repeated three times. The resulting white powder was dried under vacuum yielding 1.62 g, (87.6%) of (iBu-POSS-S-OH)(4). ^29^Si NMR (CDCl_3_) δ(ppm): –67.54[s, (–O)_3_SiCH_2_–CH], –67.88[s, (–O)_3_Si–CH_2_–CH], –70.00 [s, (–O)_3_Si–CH_2_–CH_2_]; ^1^H NMR (CDCl_3_) δ(ppm): 0.61 (d, 14H, CH–CH_2_–Si), 0.96 (d, 42H, (CH_3_)_2_CH), 0.99 (m, 2H, CH_2_–CH_2_–Si), 1.3–1.65 (m, 8H, –CH_2_-alkyl), 1.85 (m, 7H, CH–CH_3_), 2.52 (m, 2H, CH_2_–S), 2.61 (m, 2H, S–CH_2_–CH_2_–Si), 3.65 (t, 2H, CH_2_OH), 5.83-6.08 (m, CH=CH_2_). ^13^C NMR (CDCl_3_) δ(ppm): 12.30 (CH_2_–Si), 21.15(CH_2_–Si), 22.53 (CH_3_–CH), 24.44 (CH–CH_2_), 24.28, 24.9, 27.47, 28.18, 30.21, 31.37 [CH_2_–S–CH_2_(CH_2_)_4_], 61.57 (CH_2_OH). Anal. Found: C, 44.25; H, 8.24; S, 3.24, C_36_H_80_O_13_S_1_Si_8_ Calcd.: C, 44.22; H, 8.25; S, 3.27.

### 3.4. General Polymerization Procedure (5)

Hybrid materials were obtained by ROP of l,l-dilactide in the presence of Sn(Oct)2. l,l-dilactide (2.0g, 13.87 × 10^−3^ mol) and (POSS-S-OH)(1) (0.17 g, 9.96 × 10^−5^ mol), containing 7.96 × 10^−4^ mol of reactive –CH_2_OH terminated groups, were placed in a Schlenk flask connected to a high vacuum line through a Rotaflo stopcock. The reactor was evacuated for 15 min and filled with argon. Toluene (3 mL) and dioxane (1 mL) were added and the mixture was stirred with a magnetic stirrer and gently heated at 50 °C to dissolve the l,l-dilactide monomer. Tin (II) ethylhexanoate, Sn(Oct)_2_, (0.02 g, 4,9 × 10^−5^ mol) was added and the polymerization was carried out at 110 °C for 24 h. ^1^H NMR analysis of the crude hybrid (POSS-S-PLA)(5) showed 94.05% conversion of LA and SEC (MALS) analysis gave the respective values of molecular weight as *M*_n_ = 28,500, *M*_w_/*M*_n_ = 1.15. The solvents were evaporated under reduced pressure and the resulting (POSS-S-PLA) hybrid polymer was dissolved in dichloromethane and precipitated in cold methanol, filtered off, and washed several times with methanol to remove the unreacted LA and catalyst, and dried to give 1.99 g (yield 91.7%). ^29^Si NMR (CDCl_3_) δ(ppm): –68.87(s, CH_2_–Si). ^1^H NMR (CDCl_3_) δ(ppm): 0.98 (m, 2H, CH_2_–Si), 1.25–1.74 (m, CH_2_ hex and CH_3,_ PLA), 2.48 (m, 2H, CH_2_–S), 2.56 (m, 2H, Si–CH_2_–CH_2_–S), 4.11 (m, 2H, CH_2_–O–C(O)), 4.33 (q, H, CH(CH_3_)OH), 5.16 (q, H, CH(CH_3_)). ^13^C NMR (CDCl_3_) δ(ppm): 11.81 (CH_2_–Si), 16.74 (CH_3,_ PLA), 20.41[CH(CH_3_)OH], 25.32 (CH_2_–S), 31.69 (S–CH_2_), 25.93, 28.31, 28.44, 29.19 (4 × CH_2_ hex), 65.36 (CH_2_–O–C(O)), 66.58 [CH(CH_3_)OH], 69.12 (CH, PLA), 169.52 (C=O, PLA), 170.01 (CH_2_O–C(O)), 175.01 (C=O_end_), *M*_n_ (MALS) = 28,500, *M*_w_/*M*_n_ = 1.15, average length of the PLA chain *M*_n_ = 3300 (23 of monomeric units).

Bu-polylactide polymer, initiated from n-butanol in the presence of tin (II) ethylhexanoate was prepared as described above, *M*_n_(RI) = 17,800, PDI = 1.4.

## 4. Conclusions

We successfully prepared two novel types of silsesquioxane based initiators for ring opening polymerization of l,l-dilactide. They were synthesized by thiol-ene addition of 6-mercapto-1-hexanol to mono- and octavinylsilsesquioxanes. The mono- and multihydroxy silsesquioxane initiators could be effectively used for the synthesis of tailor-made biodegradable inorgano-organic star shape and linear polymers. The structure of both initiators and the respective polymer composites was proven by mass spectrometric analysis and systematic ^1^H, ^13^C and ^29^Si NMR.

## Supplementary Materials

Supplementary materials can be accessed at: http://www.mdpi.com/1996-1944/8/7/4400/s1.

## References

[1] Heyl D., Rikowski E., Hoffmann R.C., Schneider J.J., Fessner W.-D. (2010). A “Clickable” Hybrid Nanocluster of Cubic Symmetry. Chem. Eur. J..

[2] Yuan W., Liu X., Zou H., Li J., Yuan H., Ren J. (2013). Synthesis, Self-Assembly, and Properties of Homoarm and Heteroarm Star-Shaped Inorganic–Organic Hybrid Polymers with a POSS Core. Macromol. Chem. Phys..

[3] Chen P., Huang X., Zhang Q., Xi K., Jia X. (2013). Hybrid networks based on poly(styrene-co-maleic anhydride) and N-phenylaminomethyl POSS. Polymer.

[4] Zhang W., Müller A.H.E. (2013). Architecture, self-assembly and properties of well-defined hybrid polymers based on polyhedral oligomeric silsequioxane (POSS). Prog. Polym. Sci..

[5] Zhang K., Li B., Zhao Y., Li H., Yuan X. (2014). Functional POSS-Containing Polymers and Their Applications. Prog. Chem..

[6] Trastoy B., Bonsor D.A., Pérez-Ojeda M.E., Jimeno M.L., Méndez-Ardoy A., Fernández J.M.G., Sundberg E.J., Chiara J.L. (2012). Synthesis and Biophysical Study of Disassembling Nano hybrid Bioconjugates with a Cubic Octasilsesquioxane Core. Adv. Funct. Mater..

[7] Kaneshiro T.L., Wang X., Lu Z.-R. (2007). Synthesis, Characterization, and Gene Delivery of Poly-l-lysine Octa(3-aminopropyl)silsesquioxane Dendrimers: Nanoglobular Drug Carriers with Precisely Defined Molecular Architectures. Mol. Pharm..

[8] Ni C., Zhu G., Zhu C., Yao B., Kumar D.N.T. (2010). Studies on core–shell structural nano-micelles based on star block copolymer of poly(lactide) and poly(2-(dimethylamino)ethyl methacrylate). Colloid. Polym. Sci..

[9] Skaria S., Schricker S.R. (2010). Synthesis and Characterization of Inorganic-Organic Hybrid Materials Derived from Polysilsesquioxanes (POSS). J. Macromol. Sci. Part A Pure Appl. Chem..

[10] Muthukrishnan S., Plamper F., Mori H., Muller A.H.E. (2005). Synthesis and Characterization of Glycomethacrylate Hybrid Stars from Silsesquioxane Nanoparticles. Macromolecules.

[11] Lipik V.T., Widjaja L.K., Liow S.S., Abadie M.J.M., Venkatraman S.S. (2010). Effects of transesterification and degradation on properties and structure of polycaprolactone-polylactide copolymers. Polym. Degrad. Stab..

[12] Zhang W., Wang S., Li X., Yuan J., Wang S. (2012). Organic/inorganic hybrid star-shaped block copolymers of poly(L-lactide) and poly(N-isopropylacrylamide) with a polyhedral oligomeric silsesquioxane core: Synthesis and self-assembly. Eur. Polym J..

[13] Qiu Z., Pan H. (2010). Preparation, crystallization and hydrolytic degradation of biodegradable poly(L-lactide)/polyhedral oligomeric silsesquioxanes nanocomposite. Composites Sci. Technol..

[14] Hamad K., Kaseem M., Yang H.W., Deri F., Ko Y.G. (2015). Properties and medical applications of polylactic acid. Express Polym. Lett..

[15] Stanczyk W.A., Ganicz T., Gradzinska K., Kowalewska A., Kurjata J., Rozga-Wijas K. POSS-anthracycline composites. Proceedings of the International Workshop on Silicon-Based Polymers.

[16] Ma F.W., Jin Y., Zhang W.F., Zhou S.B., Ni C. (2010). Drug release properties of sodium alginate hydrophobically modified by star polylactic acid. Acta Pharm. Sin..

[17] Zhang W.-B., Li Y., Li X., Dong X., Yu X., Wang C.-L., Wesdemiotis C., Quirk R.P., Cheng S.Z.D. (2011). Synthesis of Shape Amphiphiles Based on Functional Polyhedral Oligomeric Silsesquioxane End-Capped Poly(L-Lactide) with Diverse Head Surface Chemistry. Macromolecules.

[18] Lee J.H., Jeong Y.G. (2010). Preparation and Characterization of Nanocomposites Based on Polylactides Tethered with Polyhedral Oligomeric Silsesquioxane. J. Appl. Polym. Sci..

[19] Kuo S.-W., Chang F.-C. (2011). POSS related polymer nanocomposites. Progr. Polym. Sci..

[20] Monticelli O., Cavallo D., Bocchini S., Frache A., Carniato F., Tonelotto A. (2011). A Novel Use of Ti-POSS as Initiator of L-Lactide Ring-Opening Polymerization. J. Polym. Sci. Part A Polym. Chem..

[21] Goffin A.-L., Duquesne E., Moins S., Aleksandre M., Dubois P. (2007). New organic-inorganic nanohybrids via ring opening polymerization of (di)lactones initiated by functionalized polyhedral oligomeric silsesquioxane. Eur. Polym. J..

[22] Zou J., Chen X., Jiang X.B., Zhang J., Guo Y.B., Huang F.R. (2011). Poly(L-lactide) nanocomposites containing octaglycidylether polyhedral oligomeric silsesquioxane: Preparation, structure and properties. Express Polym. Lett..

[23] Chan S.-C., Kuo S.-W., She H.-S., Lin H.-M., Lee H.-F., Chang F.-C. (2007). Supramolecular aggregations through the inclusion complexation of cyclodextrins and polymers with bulky end groups. J. Polym. Sci. Part A Polym. Chem..

[24] Cordes D.B., Lickiss P.D., Rataboul F. (2010). Recent Developments in the Chemistry of Cubic Polyhedral Oligosilsesquioxanes. Chem. Rev..

[25] Fabritz S., Hörner S., Avrutina O., Kolmar H. (2013). Bioconjugation on cube-octameric silsesquioxanes. Org. Biomol. Chem..

[26] Rozga-Wijas K., Chojnowski J. (2012). Synthesis of New Polyfunctional Cage Oligosilsesquioxanes and Cyclic Siloxanes by Thiol-ene Addition. J. Inorg. Organomet. Polym..

[27] Posner T. (1905). Beiträge zur kenntniss der ungesättigten verbindungen. II. ueber die addition von mercaptanen an ungesättigte kohlenwasserstoffe. Ber. Dtsch. Chem. Ges..

[28] Li Y., Dong X.-H., Guo H., Wang Z., Chen Z., Wesdemiotis C., Quirk R.P., Zhang W.B., Cheng S.Z.D. (2012). Synthesis of shape amphiphiles based on POSS tethered with two symmetric/asymmetric polymer tails via sequential “grafting-from” and thiol-ene “click” chemistry. ACS Macro Lett..

[29] Yu X., Zhong S., Li X., Tu Y., Yang S., von Horn R.M., Ni C., Pochan D.J., Quirk R.P., Wesdemiotis C. (2010). A giant surfactant of polystyrene-(carboxylic acid-functionalized polyhedral oligomeric silsesquioxane) amphiphile with highly stretched polystyrene tails in micellar assemblies. J. Am. Chem. Soc..

[30] Yu B., Jiang X., Qin N., Yin J. (2011). Thiol–ene photocrosslinked hybrid vesicles from co-assembly of POSS and poly(ether amine) (PEA). Chem. Commun..

[31] Clark T.S., Hoyle C.E., Nazarenko S. (2008). Kinetics analysis and physical properties of photocured silicate-based thiol-ene nanocomposites: The effects of vinyl POSS ene on the polymerization kinetics and physical properties of thiol-triallyl ether networks. J. Coat. Technol. Res..

[32] Wang Z., Li Y., Dong X.-H., Yu X., Guo K., Su H., Yue K., Wesdemiotis C., Cheng S.Z.D., Zhang W.-B. (2013). Giant gemini surfactants based on polystyrene-hydrophilic polyhedral oligomeric silsesquioxane shape amphiphiles: sequential “click” chemistry and solution self-assembly. Chem. Sci..

[33] Gao Y., Eguchi A., Kakehi K., Lee Y.C. (2004). Efficient Preparation of Glycoclusters from Silsesquioxanes. Org. Lett..

[34] Conte M.L., Staderini S., Chambery A., Berthet N., Dumy P., Renaudet O., Marra A., Dondoni A. (2012). Glycoside and peptide clustering around the octasilsesquioxane scaffold via photoinduced free-radical thiol-ene coupling. The observation of a striking glycoside cluster effect. Org. Biomol. Chem..

[35] Xu J., Li X., Cho C.M., Toh C.L., Shen L., Mya K.Y., Lu X., He C. (2009). Polyhedral oligomeric silsesquioxanes tethered with perfluoroalkylthioether corner groups: Facile synthesis and enhancement of hydrophobicity of their polymer blends. J. Mater. Chem..

[36] Hoyle C.E., Bowman C.N. (2010). Thiol-Ene Click Chemistry. Angew. Chem. Int. Ed..

[37] Lowe A.B. (2014). Thiol-ene “click” reactions and recent applications in polymer and materials synthesis: a first update. Polym. Chem..

[38] Dondoni A., Marra A. (2012). Recent applications of thiol-ene coupling as a click process for glycoconjugation. Chem. Soc. Rev..

[39] Rozga-Wijas K., Chojnowski J., Zundel T., Boileau S. (1996). Controlled synthesis of siloxane copolymers having an organosulfur group by polymerization of cyclotrisiloxanes with mixed units. Macromolecules.

[40] Li L., Xue L., Feng S., Liu H. (2013). Functionalization of monovinyl substituted octasilsesquioxane via photochemical thiol-ene reaction. Inorg. Chim. Acta.

[41] Duda A. (2012). ROP of Cyclics Esters. Mechanisms of Ionic and Coordination Processes. Polym. Sci. A Compr. Ref..

[42] Penczek S., Cypryk M., Duda A., Kubisa P., Słomkowski S. (2007). Prog. Polym. Sci..

[43] Ragheb R.T., Riffle J.S. (2008). Synthesis and characterization of poly(lactide-b-siloxane-b-lactide) copolymers as magnetite nanoparticle dispersants. Polymer.

[44] Kowalski A., Duda A., Penczek S. (2000). Kinetics and mechanism of cyclic esters polymerization initiated with tin(II) octoate. polymerization of L,L-dilactide. Macromolecules.

[45] Wyatt P.J. (1993). Light scattering and the absolute characterization of macromolecules. Anal. Chim. Acta.

[46] Libiszowski J., Kowalski A., Biela T., Duda A. (2004). Thermal stability of poly(L-lactide) prepared by polymerization of l,l-dilactide with Sn(II)-based initiators. Polimery.

[47] Blanco I., Bottino F.A., Cicala G., Cozzo G., Latteri A., Recca A. (2014). Synthesis and thermal characterization of new dumbbell shaped POSS/PS nanocomposites: Influence of the symmetrical structure of the nanoparticles on the dispersion/aggregation in the polymer matrix. Polym. Compos..

[48] Perrin D.D., Armarego W.L.F., Perrin D.R. (1980). Purification of Laboratory Chemicals.

