# Supplementary material for: Star-Shaped and Linear POSS-Polylactide Hybrid Copolymers
Source: Materials (Basel). 2015 Jul 17;8(7):4400–20. doi: 10.3390/ma8074400 (PMC5455638; doi:10.3390/ma8074400)
Supplement: Supplementary file 1 [file materials-08-04400-s001.pdf]

Supplementary Materials

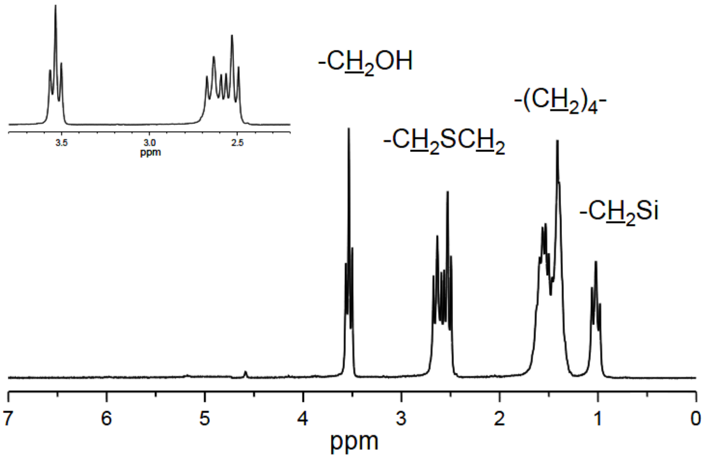

Figure S1. ^{1}H NMR(CD_3OD) spectrum of octakis-2[(6-hydroxyhexyl)thio]ethyl-octasilsesquioxane (POSS-S-OH)(**1**) obtained by thiol-ene addition of 6-mercaptokexanol-1 to octavinyloctasilsesquioxane monomer (conversion of vinyl groups 99.45%, content of functional groups, 4.66 mmol/g).

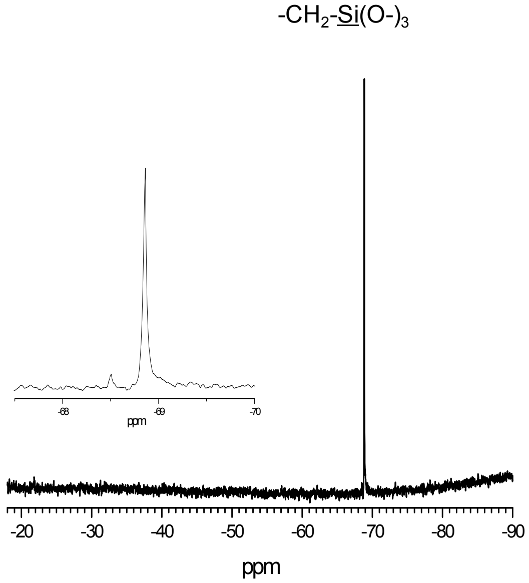

Figure S2. ^{29}Si NMR spectrum of octakis-2[(6-hydroxyhexyl)thio]ethyl-octasilsesquioxane (POSS-S-OH)(**1**) obtained by thiol-ene addition of 6-mercaptokexanol-1 to octavinyloctasil-sesquioxane (monomer conversion of vinyl groups 99.45%, content of functional groups:, 4.7 mmol/g). The spectrum was taken in CD_3OD by invgate technique.

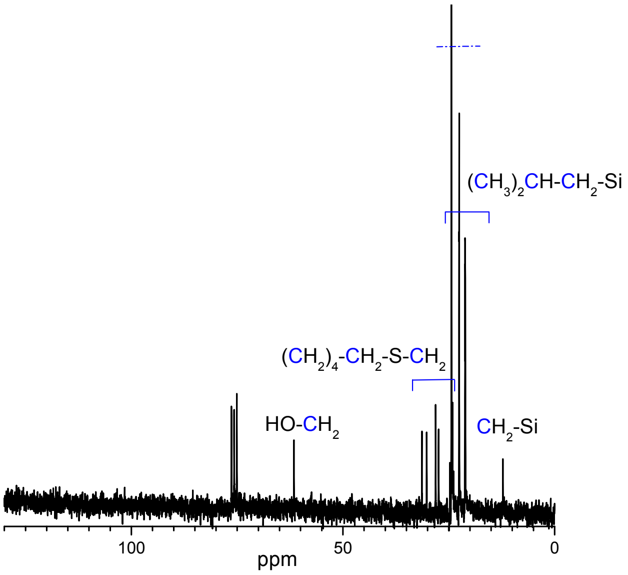

Figure S3. ^{13}C NMR ($CDCl_3$) spectrum of (iBu-POSS-S-OH)(**4**) obtained by thiol-ene addition of 6-mercaptokexanol-1 to vinylheptaisobutylooctasilsesquioxane, (conversion of vinyl groups 92.54%, content of functional groups: 1.01 mmol/g).

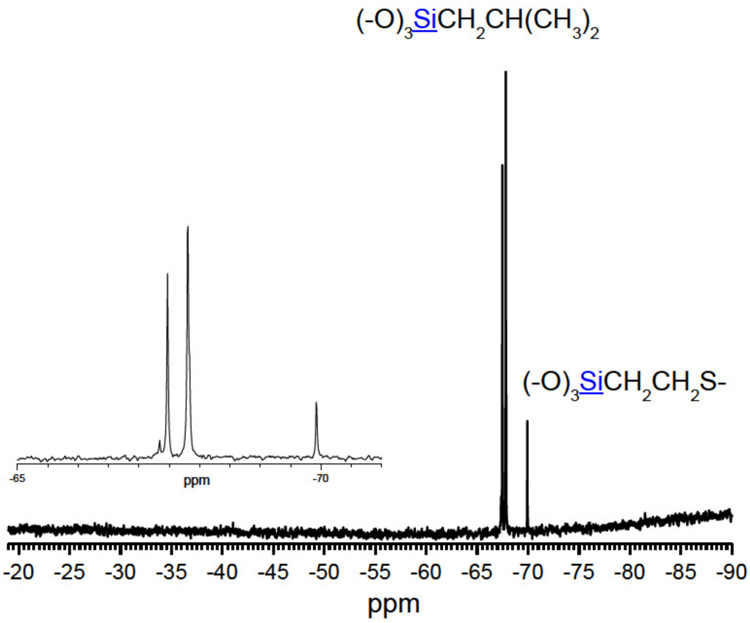

Figure S4. ^{29}Si NMR spectrum of (iBu-POSS-S-OH)(**4**). The spectrum was taken in $CDCl_3$ by invgate technique.

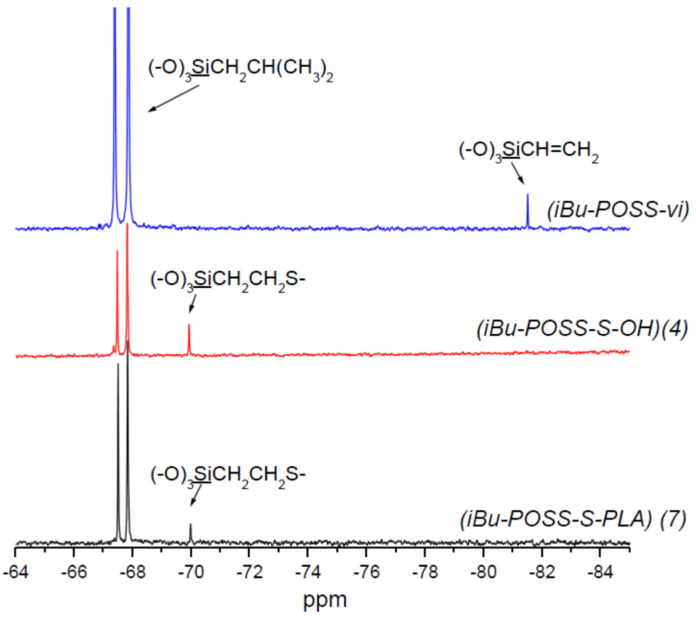

Figure S5. Comparision of the ^{29}Si NMR spectra of heptaisobuthylvinyloctasilsesquioxane (iBu-POSS-vi) (top) and (iBu-POSS-S-OH)(**4**) (middle) and (iBu-POSS-S-PLA)(**7**) polymer hybrid (bottom).

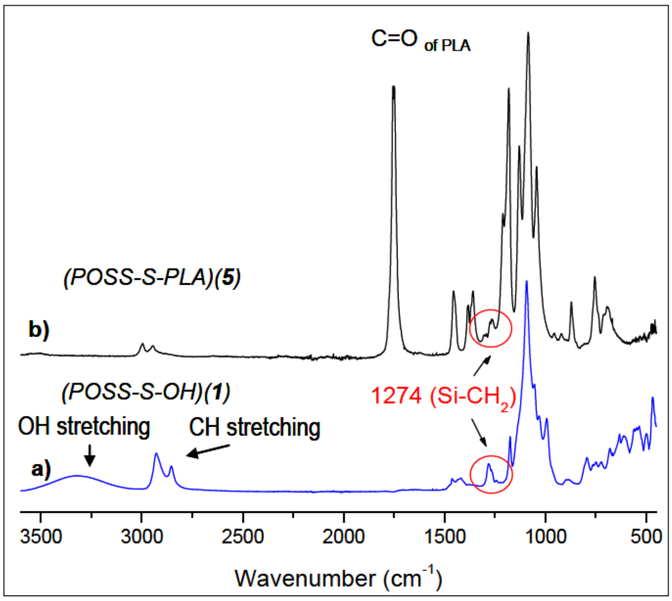

Figure S6. FT-IR spectra of the (**a**) (POSS-S-OH)(**1**) and (**b**) (POSS-S-PLA)(**5**).

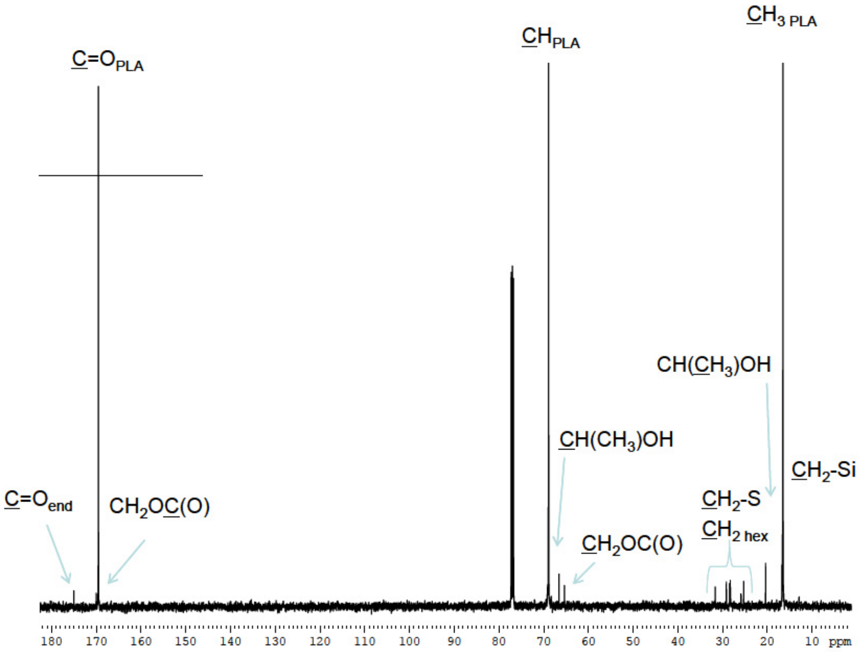

Figure S7. The ^{13}C-NMR spectrum of star-shaped (POSS-S-PLLA)(**5**) polymer hybrid. Molecular weight of hybrid M_n = 28,500, PDI = 1.15, molecular weight of polylactide chain M_n = 3300 and 8 polylactide chain per cage. The spectra was taken in $CDCl_3$ as solvent.

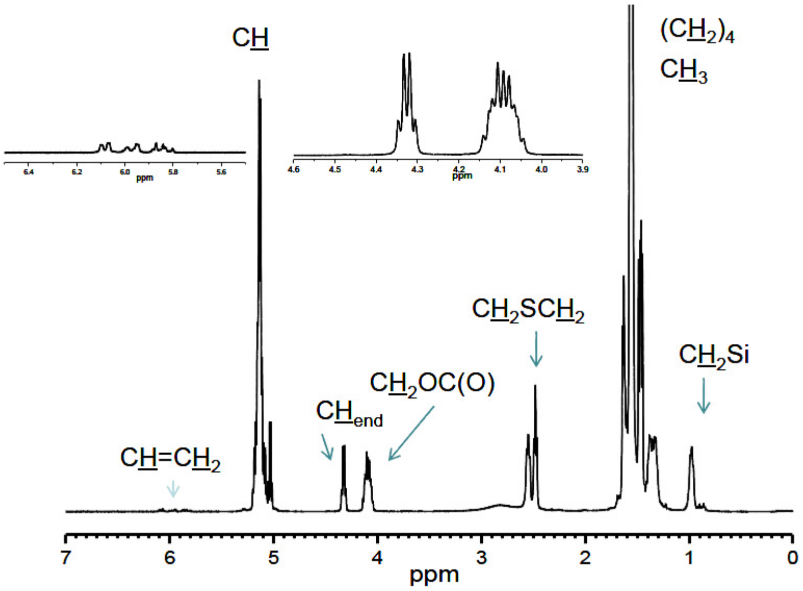

Figure S8. The ^{1}H- NMR spectrum of the star-shaped (POSS-S-PLLA)(**6**)polymer hybrid obtained by polymerization of LL-dilactide in the presence of octakis-2[(6-(hydroxyhexyl) thio]ethyl-octasilsesquioxane (**2**) and Sn(Oct)$_2$. Molecular weight of hybrid M_n = 9050, PDI = 1.07, molecular weight of polylactide chain M_n = 960 and 7.63 polylactide chain per cage. The spectra was taken in $CDCl_3$ as solvent.

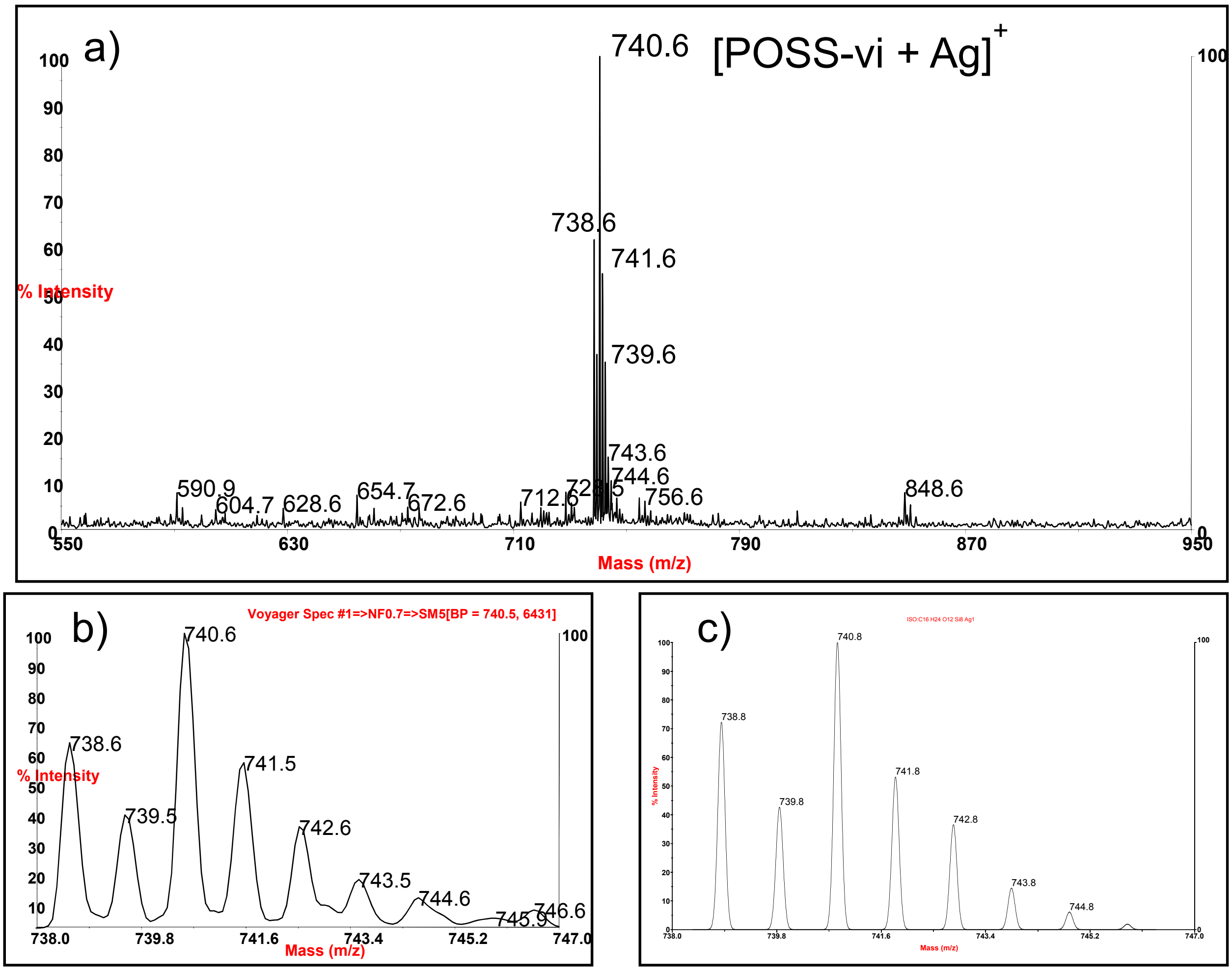

Figure S9. MALDI-TOF spectra (linear mode, AgTFA added) of the (POSS-vi), $C_{16}H_{24}O_{12}Si_8$, 633.04 (**a**), expansion of the 738-747 m/z range (**b**), simulated spectrum (**c**).
